# Supplementary material for: Establishing the proportion of severe/moderately severe vs mild cases of progressive disseminated histoplasmosis in patients with HIV
Source: PLoS Negl Trop Dis. 2022 Nov 4;16(11):e0010856. doi: 10.1371/journal.pntd.0010856 (PMC9668148; doi:10.1371/journal.pntd.0010856)
Supplement: Attachment — Submitted filename: Dear Editor (1).docx [file pntd.0010856.s001.docx]

Dear Editor, we are very grateful for the reviewer comments. You will find our responses inserted after each comment hereunder. We have also tried to improved punctuation and added a reference to back-up one of the statements.

Reviewer #1: The study design is appropriate.
The objectives are well displayed and articulated with methods.
The sample size is substantial. All subjects are from one tropical territory.
More demographic data is advisable.

*=>thank you for pointing this out. we have added some information in a new table 1 the others were renumbered. There was no significant difference between severe/moderately severe and mild.*

The authors used simple and efficient statistical analysis.
There is no concern about ethical or regulatory requirements.

Reviewer #2: The manuscript "Establishing the proportion of severe/moderately severe vs mild cases of progressive disseminated histoplasmosis in patients with HIV" is very interesting and clearly shows the difficulty in establishing the thin line between severe and moderately severe cases versus mild cases when the physician caring of any particular patient receives the patient, diagnoses HIV infection as well as histoplasmosis infection at the first contact.

This huge clinical difficulty is described in this manuscript and the methods used for this evaluation are adequated.

Therefore the study design addressed the objectives to prove the initial hypothesis tested, and used adequated analysis methods to achieve the goal.

As this study is retrospective, there are no concerns about the ethical requirements.

Due to the fact that histoplasmosis infection is not so common, even among HIV+ patients, the range of time (40 years) led to the possibility of modifications in the proposed therapy for the fungal disease, as well as HIV infection.

This is a very difficult confounder to evaluate. In order to exclude this confounder the authors used the whole number of cases through all the time to study the distribution of histoplasmosis cases relative to HIV diagnosis, and calculated the death risk difference at one month of diagnosis only for the patients diagnosed after 1999, when liposomal amphotericin B was available.
Moreover, the therapy of HIV infection evolved significantly in this time, allowing nowadays that HIV+ patients treated and with a good adherence to the therapy are protected from the acquisition of severe infections by opportunistic pathogens such as Histoplasma spp.

--------------------

**Results**


-Does the analysis presented match the analysis plan?

-Are the results clearly and completely presented?

-Are the figures (Tables, Images) of sufficient quality for clarity?


Reviewer #1: The results are well exposed.
Tables 1 and 2 could be more reader-friendly if they had horizontal stripes individualizing the lines.

*=>ok thanks we amended the tables we hope this is what was expected from us.*

Images are well conceived.

Reviewer #2: The results are clearly presented and described in the text, and the figures are adequately clear to the readers helping them to understand quickly the data presented in the manuscript.

--------------------

**Conclusions**


-Are the conclusions supported by the data presented?

-Are the limitations of analysis clearly described?

-Do the authors discuss how these data can be helpful to advance our understanding of the topic under study?

-Is public health relevance addressed?


Reviewer #1: Data presented supports conclusions from an appealing point of view and with a simple and nonordinary focus.
Discussion competently addresses the results for public health, sometimes with some "not so suitable" extrapolations, like cost calculations, which were not researched in this study.

*=>yes we agree. we have specified that these are very coarse and are not proper cost effectiveness study*
The description of study limitations should be more precise. The authors could reserve more words for this.

*=> thank you we have expanded notably about the retrospective nature and long time span which may entail many sources of variation. We hope it is better. We have also tried to be quite clear about the limitations of costing exercises.*

Reviewer #2: The conclusions are supported by the data, and the discussion of the results are adequate and helpful to explain to the reader the tough decisions presented to the clinical physician caring the HIV patients presenting with histoplasmosis.

--------------------

**Editorial and Data Presentation Modifications?**


 Reviewer #1: The authors inform that data will be avaiable for reasonable requests. Editors could consider asking about wich conditions are they. Maybe PLoS could solve them, or at least provide enhanced guidance to the scientists.
Tables 1 and 2 could be more reader-friendly if they had horizontal stripes individualizing the lines.

Reviewer #2: No, the text is very well written and clear, and the presentation of data adequate to help the reader in the decision of therapy when he/she is presented to similar cases.

--------------------

**Summary and General Comments**


Reviewer #1: The topic under study received a concrete contribution from this paper. Histoplasmosis in the setting of HIV/AIDS needs more knowledge about it, particularly in the tropics.
Statistical analyses are well explained and simplified to the reader, which is an excellent marker. The idea of showing attributable fractions is a simple way to clarify data to the reader.
Minor reviews are addressed in anterior fields:
- Presentation of more detailed demographic data;

*=>we added a table*
- Minimize atrapolations about cost;

*=>we criticized these extrapolations and emphasized they were crude and were not proper cost effectiveness studies so there is no ambiguity*
- A more careful discussion about study limitations.
*=>we have expanded*
I hope I have helped and salute the authors for the well-qualified study.

*=>many thanks*! We hope the changes are ok

Reviewer #2: Summarizing the review, in my point of view this manuscript is clinically very interesting and adequately executed, allowing it to be published at PLOS NTD.
